# Supplementary material for: HIVprotI: an integrated web based platform for prediction and design of HIV proteins inhibitors
Source: J Cheminform. 2018 Mar 9;10:12. doi: 10.1186/s13321-018-0266-y (PMC5845081; doi:10.1186/s13321-018-0266-y)
Supplement: Supplementary file 2 — Additional file 2. Source code of HIVProtI web server. [file 13321_2018_266_MOESM2_ESM.zip › HIVprotI_Source-code/hivprotI/weka-3-6-6/doc/weka/gui/visualize/AttributePanelEvent.html]

AttributePanelEvent


---


|  |  |  |
| --- | --- | --- |
| **Overview** \| **Package** \| **Class** \| **Tree** \| **Deprecated** \| **Index** \| **Help** \| **Weka's home** | |  |
| **PREV CLASS**   **NEXT CLASS** | **FRAMES**    **NO FRAMES**     **All Classes** |  |
| SUMMARY: NESTED \| FIELD \| CONSTR \| METHOD | DETAIL: FIELD \| CONSTR \| METHOD |  |


---


## weka.gui.visualize Class AttributePanelEvent

```
java.lang.Object
  weka.gui.visualize.AttributePanelEvent
```

---

``` public class AttributePanelEvent extends java.lang.Object ```

Class encapsulating a change in the AttributePanel's selected x and y
attributes.

**Version:**
:   $Revision: 1.4 $

**Author:**
:   Mark Hall (mhall@cs.waikato.ac.nz)

---

| **Field Summary** | |
| --- | --- |
| `int` | `m_indexVal`             The index for the new attribute |
| `boolean` | `m_xChange`             True if the x selection changed |
| `boolean` | `m_yChange`             True if the y selection changed |


**Constructor Summary**

`AttributePanelEvent(boolean xChange,
boolean yChange,
int indexVal)`
  
          Constructor


**Method Summary**

**Methods inherited from class java.lang.Object**

`equals, getClass, hashCode, notify, notifyAll, toString, wait, wait, wait`

**Field Detail**

### m\_xChange

```
public boolean m_xChange
```

:   True if the x selection changed

---


### m\_yChange

```
public boolean m_yChange
```

:   True if the y selection changed

---


### m\_indexVal

```
public int m_indexVal
```

:   The index for the new attribute


**Constructor Detail**

### AttributePanelEvent

```
public AttributePanelEvent(boolean xChange,
                           boolean yChange,
                           int indexVal)
```

:   Constructor

    **Parameters:**: `xChange` - true if a change occured to the x selection: `yChange` - true if a change occured to the y selection: `indexVal` - the index of the new attribute


---


|  |  |  |
| --- | --- | --- |
| **Overview** \| **Package** \| **Class** \| **Tree** \| **Deprecated** \| **Index** \| **Help** \| **Weka's home** | |  |
| **PREV CLASS**   **NEXT CLASS** | **FRAMES**    **NO FRAMES**     **All Classes** |  |
| SUMMARY: NESTED \| FIELD \| CONSTR \| METHOD | DETAIL: FIELD \| CONSTR \| METHOD |  |


---
